# Evolutionarily Stable Coevolution Between a Plastic Lytic Virus and Its Microbial Host

**DOI:** 10.3389/fmicb.2021.637490

**Published:** 2021-05-20

**Authors:** Melinda Choua, Michael R. Heath, Juan A. Bonachela

**Affiliations:** ^1^Marine Population Modeling Group, Department of Mathematics and Statistics, University of Strathclyde, Scotland, United Kingdom; ^2^Department of Ecology, Evolution and Natural Resources, Rutgers University, New Brunswick, NJ, United States

**Keywords:** phage (bacteriophage), virus modeling, lysis, viral latency, *E. coli*, T phage, host-virus interactions

## Abstract

Hosts influence and are influenced by viral replication. Cell size, for example, is a fundamental trait for microbial hosts that can not only alter the probability of viral adsorption, but also constrain the host physiological processes that the virus relies on to replicate. This intrinsic connection can affect the fitness of both host and virus, and therefore their mutual evolution. Here, we study the coevolution of bacterial hosts and their viruses by considering the dependence of viral performance on the host physiological state (viral plasticity). To this end, we modified a standard host-lytic phage model to include viral plasticity, and compared the coevolutionary strategies emerging under different scenarios, including cases in which only the virus or the host evolve. For all cases, we also obtained the evolutionary prediction of the traditional version of the model, which assumes a non-plastic virus. Our results reveal that the presence of the virus leads to an increase in host size and growth rate in the long term, which benefits both interacting populations. Our results also show that viral plasticity and evolution influence the classic host quality-quantity trade-off. Poor nutrient environments lead to abundant low-quality hosts, which tends to increase viral infection time. Conversely, richer nutrient environments lead to fewer but high-quality hosts, which decrease viral infection time. Our results can contribute to advancing our understanding of the microbial response to changing environments. For instance, both cell size and viral-induced mortality are essential factors that determine the structure and dynamics of the marine microbial community, and therefore our study can improve predictions of how marine ecosystems respond to environmental change. Our study can also help devise more reliable strategies to use phage to, for example, fight bacterial infections.

## Introduction

Cell size is a key trait for microbial systems. Replication rates and physiological traits are generally correlated with cell size, both within and across species (Litchman et al., 2007). For virus-bacteria interactions, the size of the host is particularly important. In this paper, we focus on viral infections as an evolutionary selective pressure for host body size.

The adsorption of viruses to host cells depends on a combination of multiple factors. Host size influences the probability of host-virus encounter (Delbrück, 1940; Rabinovitch et al., 2002), and the density of host receptors to which the virus attaches (Schwartz, 1976; Berg and Purcell, 1977). In addition, the size of the host influences the surface-to-volume ratio, which triggers additional consequences e.g., increasing cell size decreases the diffusion flux per unit cell volume of external nutrient into the cell, but increases internal diffusion rates (Gallet et al., 2017). Both external and internal diffusion rates affect the mobility of intracellular resources used by the host. The latter, among other factors influenced by cell size such as resource uptake, affects the host physiological state. Here, we use growth rate as a proxy for host physiological state because it captures a diversity of intracellular indicators (Bremer and Dennis, 2008). In turn, the host physiological state influences the performance of the viral infection (Calendar and Abedon, 2005) and thus the dynamics of both viral and bacterial populations (Choua and Bonachela, 2019).

Viral infection starts when the virus encounters a host and becomes irreversibly attached onto a specific receptor at the host surface, with the frequency of successful encounters defining the adsorption rate (Calendar and Abedon, 2005). Once adsorbed, the virus perforates the cell’s membrane and inserts its genome into the host cytoplasm, which triggers the biosynthesis of viral genome and proteins by the host machinery. The host machinery (ribosomes, ATP, etc.) is therefore monopolized by the virus to transcribe proteins that will constitute the viral offspring. The time between attachment and the assembly of the first virion defines the eclipse period, and the number of virions assembled per unit time is the maturation rate, both depending mainly on the host physiological state. The lytic cycle ends with the lysis of the host and the consequent release of virions into the environment (Abedon et al., 2003). The time between adsorption and host lysis, and number of virions released to the environment per infection, define the latent period and burst size, respectively. For a given host growth rate, longer latent periods typically lead to larger burst sizes (Abedon, 1989; Gnezda-Meijer et al., 2006; Wang, 2006).

Experimental work has shown that these key viral traits are not fixed, but change with the host physiological state (Rabinovitch et al., 2002; You et al., 2002; Birch et al., 2012; Golec et al., 2014): eclipse and latent periods decrease, and maturation rate and burst size increase, with host growth rate. Thus, intracellular changes in the host (effectively the phage’s environment) lead to either active or passive responses by the virus, which are typically referred to as viral phenotypic plasticity (Abedon et al., 2001; Choua and Bonachela, 2019).

Viral plasticity has been shown to have important ecological and evolutionary implications for host-phage systems (Thyrhaug et al., 2003; Weitz and Dushoff, 2008; Wang and Goldenfeld, 2010; Edwards and Steward, 2018; Choua and Bonachela, 2019; Choua et al., 2020). It is yet to be determined, however, how viral plasticity affects the evolution of the host and, more specifically, the evolution of a trait so important for bacteria-virus interactions as host body size. Larger bacterial cell sizes lead to higher maximal growth rates (Gallet et al., 2017), but typically decrease uptake affinity (Wirtz, 2002) and render the host more susceptible to viral adsorption (Delbrück, 1940). Accounting for viral plasticity, a host with a higher growth rate will not only replicate faster but will also facilitate viral replication (i.e., the virus will release more virions in a shorter time) (Choua and Bonachela, 2019). This change, in turn, may alter the selection pressure on e.g., the viral latent period, as an increase in infection time enables the release of more virions but decreases the opportunities of subsequent infections by the new offspring (Choua and Bonachela, 2019). Here, we aim to understand from an evolutionary viewpoint how host and virus affect each other in different environments, with emphasis on how viral plasticity might affect their coevolution.

Bacteria-phage coevolution has previously been studied in the context of an “arms race” (Morgan and Koskella, 2011), e.g., using theoretical systems where the host evolves resistance by changing the receptors used by the virus for attachment at a fitness cost, and the phage responds to this resistance by adapting its tail fiber to overcome the receptor changes (Weitz et al., 2005; Menge and Weitz, 2009). These studies showed that, for example, coevolution maintains phenotypic and genetic diversity within microbial communities (Koskella and Brockhurst, 2014). However, none of these studies have considered how the presence of an inherently plastic virus affects this coevolution.

To fill this knowledge gap, here we use a standard host-lytic phage model that we modified to include viral plasticity. To implement coevolution, we used a numerical ecoevolutionary framework that focuses on host size and viral latent period as representative evolving traits. With this framework, we compare the traits that emerge under coevolution to those emerging when host evolves without virus, and to those obtained in previous work when host does not evolve but virus does (Choua and Bonachela, 2019). Theoretical studies like ours can help understand and predict the coevolutionary dynamics of host and phage, and therefore contribute to a wide variety of fields, from increasing the effectiveness and safety of phage therapy to improving the reliability of biogeochemical models, since bacterioplankton cell size plays a central role in the prediction of the response of marine ecosystems to climate change (Finkel et al., 2009).

## Materials and Methods

### Model Description

To represent the ecological dynamics of the host- phage system, we used a well-known model that considers explicitly the delay between infection and lysis (Levin et al., 1977). This model has been ecologically validated by experiments, and proven to provide evolutionarily meaningful outcomes (Bonachela and Levin, 2014). The model keeps track of the dynamics of a generic nutrient (*N*), uninfected hosts cells (*C*), infected hosts (*I*) and extracellular viruses (*V*). The environmental conditions are set up to those of a chemostat, i.e., a well-stirred controlled environment in which the host and phage encounter each other randomly. Because we assume that, once a host is infected, its machinery is devoted to viral production, infected hosts do not replicate and therefore the host’s generation time does not constrain the viral latent period. We further consider that bacteria grow in the limited volume of the chemostat, and thus system-size effects can trigger competition for space, light, or other resources not explicitly modeled here that can limit/hinder population growth. This crowding effect can be implemented through a density-dependent term, in our case a quadratic loss term that only affects free hosts as uptake and growth stop at infection.

Thus, assuming that a host phenotype *i* is characterized solely by its size (i.e., all host types are phenotypically identical except for their size and consequently any trait related to it), and a viral phenoptype *j* is characterized by its latent period, the equations for the dynamics of the different phenotype populations and the nutrient are given by:

(1) $$\frac{d\,N}{d\,t}=w\,\left(N_{0}-N\right)-\frac{1}{Y}\,\sum_{i}\mu_{i}\,\left(N\right)\,C_{i}$$

(2) $$\frac{d\,C_{i}\,\left(t\right)}{d\,t}=\mu_{i}\,C_{i}-k_{i}\,C_{i}\,\sum_{j}V_{j}-w\,C_{i}-\alpha \,C_{i}\,\sum_{n}C_{n}$$

(3) d⁢Ij⁢(t)d⁢t=∑iki⁢Ci⁢Vj-∑iki⁢Cit-Lj⁢Vjt-Lj⁢e-w⁢Lj-w⁢Ij

(4) d⁢Vj⁢(t)d⁢t=Bj⁢μi⁢∑iki⁢Cit-Lj⁢Vjt-Lj⁢e-w⁢Lj-∑iki⁢Ci⁢Vj-(m+w)⁢Vj

(see definitions of symbols and units in Table 1). The first equation represents the dynamics of the concentration of the most limiting nutrient within the chemostat, with an inflow and outflow of nutrient (first term) and the nutrient uptake by all possible host phenotypes present in the chemostat at time t (second term). For the sake of concreteness, we assume that carbon is the most limiting nutrient. Note that we assume the yield factor *Y* (i.e., how much the host population can grow per unit of resource) to be the same for all host phenotypes. Equation (2) describes the growth of each host phenotype population, *C*_*i*_, as a result of reproduction (first term), infection by the different viral phenotypes present in the chemostat at time *t* (second term), bacterial loss due to dilution (third term), and population growth slowdown due to crowding (fourth term). Equation (3) keeps track of the cells infected by viral phenotype *j* (equally, the number of intracellular viruses, as we consider that a host can be infected by only a single viral individual, i.e., there is no superinfection). The first term represents the new infected individuals resulting from the adsorption of that specific *j* viral phenotype. Infected cells disappear during dilution (last term) or due to the lysis of the cells that became infected exactly one latent period (*L_j*) in the past (second term, where *e*^−*wL*_*j*_^ is the probability for infected cells to survive dilution during the latent period). The lysis of these infected cells releases new free phage for viral phenotype *j* (first term in Eq. (4), with *B* representing the burst size, see below). This pool of free phage then decreases due to phage adsorption (second term), and natural mortality and dilution (last term).

**TABLE 1:** Symbols for variables and parameters used 1in the model.

Symbol	Description	Units	Value	References
N	Dissolved inorganic nutrient concentration	mol l^–1^	Ecological variable, Eq. 1	Levin et al., 1977, for the equations
C	Non-infected-host concentration	cell l^–1^	Ecological variable, Eq. 2	
V	Free virus concentration	cell l^–1^	Ecological variable, Eq. 3	
μ	Non-infected-host population growth rate	d^–1^	Ecological variable, Eq. 4	Monod, 1949, for the equation
**Host parameters/traits**				
r	Equivalent spherical radius	μm	Evolutionary variable	Loferer-Krößbacher et al., 1998
μ_*max*_	Maximum host population growth rate	d^–1^	Eq. 6	Gallet et al., 2017
c, h	Parameters Eq. 6	—	*c* = 0.33 *h* = 3.8	Gallet et al., 2017
K_*ref*_	Half-saturation constant for μ_*max*_ = 0	mol l^–1^	3.05 × 10^–8^	Wirtz, 2002
μ_*ref*_	Asymptotic μ_*max*_ for K_*n*_→ ∞	d^–1^	32.4	Wirtz, 2002
K_*n*_	Half-saturation constant for growth	mol	Eq. 7	Wirtz, 2002
Y	Yield parameter	cell mol^–1^	9 × 10^13^	Choua and Bonachela, 2019
μ_*max, experiment*_	Maximum growth rate in the experiment	d^–1^	40.8	You et al., 2002
α	Parameter of crowding effect	l d^–1^ cell^–1^	0–12 × 10^–7^	Sensitivity analysis
**Viral parameters/traits**				
D	Diffusion of viral particle	m^2^ s^–1^	4.3132 × 10^–12^	Calculated using Stokes-Einstein expression
m	Viral decay rate	d^–1^	0.09	De Paepe and Taddei, 2006
k	Adsorption rate	l virus^–1^ d^–1^	4π D Conv_3_ r	Delbrück, 1940
E(μ)	Eclipse period	d	Eq. 8	Choua and Bonachela, 2019
M(μ)	Maturation rate	virions d^–1^	Eq. 9	Choua and Bonachela, 2019
L	Latent period	d	Evolutionary variable	
B	Burst size	virions cell^–1^	*B* = M (L-E)	Wang, 2006
**Chemostat parameters**				
w	Chemostat dilution rate	d^–1^	1–30	Ranges set by Eq(7) and range for *r*
N_0_	Dissolved inorganic nutrient supply concentration	mol l^–1^	9 × 10^–5^	Sensitivity analysis
**Conversion constants**				
Conv_1_	Constant to convert from (ml) to (μm^3^)	μm^3^ml^–1^	10^–12^	—
Conv_2_	Constant to convert from (hour^–1^) to (d^–1^)	hour d^–1^	24	—
Conv_3_	Constant to convert from (m^3^ s^–1^) to (l d^–1^)	l s d^–1^ m^–3^	86,400 × 10^3^	—

We assume that free hosts grow according to the classic Monod equation (Monod, 1949):

(5) $$\mu_{i}\,\left(N\right)=\mu_{m\,a\,x}\,\left({\text{r}}_{\text{i}}\right)\,\text{N}/\left(N+K_{n}\,\left(\mu_{\max}\right)\right)$$

where μ_*max*_(r_i_) is the maximum growth rate, and K_n_(μ_*max*_) (half saturation constant for growth) is inversely correlated with the affinity of the uptake/growth machinery for this nutrient. As explained below, both traits depend on the host’s cell size, r_i_ (Chien et al., 2012).

We use this model to represent host and phage coevolution by letting the host size and viral latent period evolve. We implement evolution using a genetic algorithm in which the phenotype with the highest probability to be selected for mutation (i.e., the one with the highest relative abundance in the system) generates a new mutant phenotype. A new host phenotype is identical to the parental cell except for its radius, chosen randomly from a normal distribution centered on the parental value and with a standard deviation of 0.1 microns. Similarly, a new viral phenotype is identical to the parent except for the value of the latent period, chosen randomly within a normal distribution centered on the parental value with a standard deviation of 10^−3^ days (narrowed to 10^−4^ days for the last 5,000 days of the simulation, when the algorithm is expected to have reached the vicinity of the evolutionary steady state). This leads to the addition of a new population every time a host mutant and/or a virus mutant join the system (Menge and Weitz, 2009; Choua and Bonachela, 2019). Note that, here, any viral phenotypes can infect any of the host phenotypes (i.e., generalist virus). Although phage are relatively specific (i.e., virus adsorbs to only a limited subset of the total bacteria), they have been shown to become generalist when coevolving with their host (Hall et al., 2011).

### Host Trait Set

For most microorganisms, size is a master trait because it affects most aspects of their life cycle (Litchman et al., 2007). Although the metabolic theory of ecology states that the growth rate of microbes decreases with increasing body size (Brown et al., 2004), recent experimental data showed that maximal growth rates tend to increase with body size for organisms smaller than six microns (Gallet et al., 2017; Ward et al., 2017). These data revealed a key trade-off between rates of resource acquisition and the rate of internal metabolism (i.e., μ_*max*_), which suggests different limiting factors for small and large organisms. For small cells such as bacteria, molecular transit time inside the cell can be the limiting factor whereas uptake rate limits larger cells (Gallet et al., 2017); also, the rate at which internal quotas are replenished by nutrient uptake limits smaller cells but nutrient conversion into biomass limits larger cells (Ward et al., 2017).

Here, we choose size as single trait characterizing the host, and use allometries to calculate the rest of trait values that are relevant for host dynamics. We specifically focus on *Escherichia coli* as host, but our approach can be generalized to any bacterium. Existing experimental work provides an allometric expression linking the radius, r, of the bacterium (assumed spherical) and its maximum potential for growth, μ_*max*_ (Gallet et al., 2017; Shestopaloff, 2016):

(6) μm⁢a⁢x⁢(ri)=Conv2⁢ 10c⁢l⁢o⁢g10⁢(4⁢π⁢Conv1⁢ri3/3)+h

(see Table 1 for symbols and units) where the c and h parameters determine how steeply the maximum growth rate increases with r_i_. In turn, the two Monod parameters are positively correlated with each other, which determines a relationship that can be mathematically expressed through the following function (Wirtz, 2002):

(7) $$K_{n}\,\left(\mu_{m\,a\,x}\right)=K_{r\,e\,f}\,e^{\mu_{m\,a\,x}\,\left({\text{r}}_{\text{i}}\right)/\left[\mu_{r\,e\,f}-\mu_{m\,a\,x}\,\left({\text{r}}_{\text{i}}\right)\right]}$$

where μ_*ref*_ represents the asymptotic maximal growth rate for an infinitely high *K_n* and *K*_*ref*_ represents half-saturation constant at μ_*max*_=0. Other forms for this relationship have previously been shown not to affect qualitatively the ecological predictions of this model (Choua et al., 2020). Note that Eq.(7) together with Eq.(6) entail that nutrient affinity declines as cell size increases.

### Phage Trait Set and Plasticity

We focus here on the T-phage subfamily, which infects *E. coli* through receptors that occupy up to 75% of the cell surface (Nikaido and Vaara, 1985). The latter allows us to approximate, for simplicity, that all collisions lead effectively to adsorption (Delbrück, 1940; Schwartz, 1976; Berg and Purcell, 1977). Importantly, the size of the host affects the viral adsorption rate, which has been represented in the past using the linear function k_i_=4r_i_*π*D (Delbrück, 1940), where D is the diffusion coefficient of the phage.

Although the exact factors that determine the latent period, L_j_, are unknown, evidence points to the so-called holin gene as the responsible of the timing of lysis (Young and Bläsi, 1995; Wang et al., 2000; Ramanculov and Young, 2001; White et al., 2011). This link between L_j_ and the holin gene justifies the characterization of phenotypes using the latent period as an evolving trait. Because this timing is typically smaller than the time needed to deplete the host resources involved in virion production (Rabinovitch et al., 1999; Wang, 2006), we assume that the burst size is only limited by the number of phage that has been assembled between the end of the eclipse period and lysis. This assumption is usually represented by the linear function *B*=M(L−E) (Wang, 2006). We consider here that E and M, respectively, the eclipse period and maturation rate, depend on the physiological state of the host (represented by the host growth rate, μ_*i*_) through the following data-informed expressions (Choua and Bonachela, 2019):

(8) $$E\,\left(\mu_{i}\right)=E_{\infty }+E_{0}\,e^{-\alpha_{E}\,\mu_{i}/\mu_{\max,\mathrm{experiment}}}$$

(9) $$M\,\left(\mu_{i}\right)=\frac{M_{\infty }}{1+e^{\alpha_{M}\,\left(\frac{\mu_{i}}{\mu_{\text{max},e\,x\,p\,e\,r\,i\,m\,e\,n\,t}}-M_{0}\right)}}$$

In short, *E* decreases and *M* increases with the host growth rate. Both functional forms show a plateau at high growth rates, which reflects the physiological limits of the host machinery to synthesizing proteins (Choua and Bonachela, 2019). *E*_∞_ and *E_0* determine *E* for very low growth rates, α_*E*_ determines the slope of the (decreasing) function, and μ_*max, experiment*_ is the maximal growth rate that the host reached in the experimental data used to deduce Eqs. (8)–(9); *M*_∞_ represents the upper plateau of the increasing sigmoid, α_*M*_ how steeply *M* reaches it, and *M_0* the midpoint of the function. Finally, Eq. (8) shows a finite value for *μ*→0, which represents the possibility of viral reproduction at very low host growth rates, observed experimentally (Golec et al., 2014). Alternatively, for such extreme conditions the virus may switch from lytic to temperate mode (e.g., lysogeny). Although such change in viral strategy can certainly influence the coevolution of the system, here we focused on obligate-lytic viruses since the main plastic traits above are linked to cell lysis. All together leads to the following plastic representation of the burst size for viral phenotype *j* infecting host *i*:

(10) $$B_{j}\,\left(\mu_{i}\right)=M\,\left(\mu_{i}\right)\,\left(L-E\,\left(\mu_{i}\right)\right)$$

For each host phenotype *i*, the viral traits [i.e., E⁢(μni), M⁢(μni), and thus *B_j*] are adjusted at each integration step to follow updates in the host growth rate. In contrast, models that neglect viral plasticity use fixed values for *E* and *M*, obtained from experiments that standardly set optimal conditions for the host (Abedon et al., 2001). In consequence, the associated *E*=*E*_*non*_ and *M*=*M*_*non*_ reflect the performance of the host machinery at the maximum growth rate expected for the particular phenotype, i.e., at μ_*i*_=μ_*max*_(r_i_). These values are thus host-specific: because different host phenotypes/strains show different sizes and therefore different μ_*max*_, *E*_*non*_ and *M*_*non*_ must follow accordingly. As we could not find values for *E* and *M* for all the different host sizes used in our simulations, we estimated those values as *E*_*non*_(*r*_*i*_)=*E*[μ_max_(*r*_*i*_)] and *M*_*non*_(*r*_*i*_)=*M*[μ_max_(*r*_*i*_)]. In consequence, the burst size for the non-plastic virus follows here the expression:

(11) $$B_{n\,o\,n}=M_{n\,o\,n}\,\left(r_{i}\right)\,\left(L-E_{n\,o\,n}\,\left(r_{i}\right)\right)$$

### Parametrization and Analysis

We use Matlab^®^ to integrate numerically these equations under different environmental conditions (i.e., different values of *w* and N_0_) and, due to the limited amount of information available about the (broadly defined) crowding strength, we also vary the parameter α in a range commensurate with the rest of terms in Eq.(2). Specifically, we try α = 0 (i.e., no crowding), α=10^−6^lcell^−^1d^−1^, and 10^−7^cell^−1^d^−1^.

Thus, during 10^4^ simulated days (enough for the system to reach stationarity), multiple hosts compete for the common nutrient (bottom-up regulation) while experiencing the mortality exerted by the viral populations (top-down pressure), which in turn compete for the available hosts. In our system, host size can evolve between 0.3 to 1.1 μm, range that provides trait values usually observed for *E. coli* (i.e., host volume, μ_*max*_, and *K_n* are compatible with experimental observations) (Schulze and Lipe, 1964; Loferer-Krößbacher et al., 1998; Füchslin et al., 2012). On the other hand, the viral latent period can evolve between a realistic maximal value for T-phage of 2 days and a certain minimal value. This minimal value is calculated based on the host phenotype that shows the highest growth rate (i.e., the smallest *E*) among all the host mutants, which ensures a minimal latent period bigger than any eclipse period in the system. The latter is necessary because we assume that any viral phenotype can infect any available host. We also consider that both host and virus mutate at similar times. Increasing the viral mutation rate to up to 5 times that of the host did not change the probability of coexistence nor the number of functional combination (results not shown), and therefore we set the less-computationally-expensive limit of equal timing for both host and viral mutations.

With this model and constraints, and starting from a pair of host-virus phenotypes with (*L*,*r*) randomly chosen within the ranges above, mutation and selection enable the stochastic exploration of the phenotypic space until, eventually, a combination of phenotype maximizing fitness for the host and virus emerges: the Evolutionary Stable Strategy (ESS) (*L*_*ESS*_,r_*ESS*_). Due to the stochastic character of the simulation, we run up to 500 replicates for each combination of *w*,N_0_, and α, in order to find the ESS as the average of the trait values emerging over replicates. We further compare the emergent ESS for both plastic and non-plastic viruses, running for the latter the non-plastic version of the model (Eq. 11).

Out of the many replicates, we select the cases that show coexistence between host and virus at the end of the simulation, and reject those that lead to host and/or viral extinction. Moreover, in order to focus on the evolutionarily stable values of the evolving traits, we retain only the replicates for which a true dominant phenotype can be discerned. Specifically, we label as dominant the phenotype such that its abundance represents more than 75% of the total mutant community (i.e., both CESSst/∑iCist and VESSst/∑jVjst above 0.75, where the subscript “st” refers to the stationary state obtained by averaging the 20 last days of the simulation).

We then analyze how these (L_ESS_,r_ESS_) combinations vary as w, N_0_, and α change. For each case, we compare the (L_ESS_,r_ESS_) for the plastic case to those obtained for the non-plastic description of the system. Finally, we compare (i) the L_ESS_ emerging from coevolution to the analytical expression provided by Choua and Bonachela (2019) for a system where only the virus evolves; and (ii) the r_ESS_ emerging from coevolution to the ESS obtained in a system where bacteria evolve in the absence of viruses.

Note that extreme conditions deterministically result in a number of useable combinations (*L*_*ESS*_,*r*_*ESS*_) that is lower than our original number of replicates. For example, at low dilution rates, virus and host cannot coexist as the associated low growth rate eventually leads to the extinction of the host (followed by the extinction of virus); at high dilution rates, viral mortality is very high and the viral population goes to extinction, which allows the host to thrive alone.

## Results

### Host Evolving Without Virus

**FIGURE 1:**
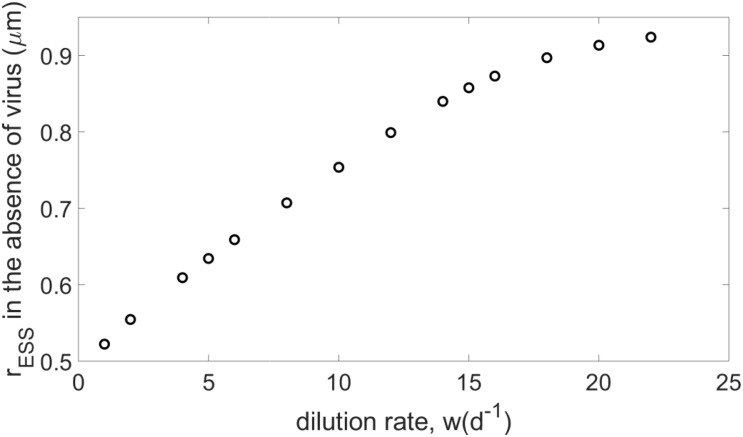
Evolutionary stable strategies (ESS) when the host evolves in the absence of the virus, for *N*_0_=10^−5^*moll*^−*l*^ and α=10^−8^*lcell*^−1^*d*^−1^, and different dilution rates.

**FIGURE 2:**
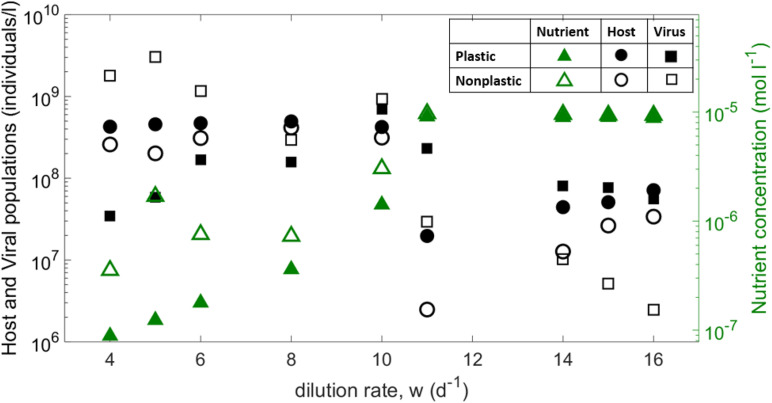
Median across replicates for the host, viral and nutrient concentrations averaged over the last 20 days of each replicate, as a function of the dilution rate. Note that small dilution rate led to wide oscillations [e.g., for *w* = 4 *d*^−1^ in the plastic case *N* varies between10^−8^ and 10^−5^*moll*^−1^].

In a simple system where the virus and crowding effect are neglected (i.e., *V*=α=0), both the ecological and evolutionary dynamics of the host can be assessed analytically (see Supplementary Appendix A). The analytical results show that the ESS for the host presents a size that minimizes nutrient consumption, in agreement with classic competition theory (Tilman, 1982). On the other hand, in cases where the virus is absent but the crowding effect is considered (i.e., *V*=0,α≠0), the evolutionary stationary state of the host can be calculated numerically using the genetic algorithm described above. As the dilution rate increases, the emerging host size increases with the dilution rate and eventually saturates (see Figure 1). Because the dilution rate is positively correlated with nutrient concentration (see Figure 2), increasing *w* leads to bigger hosts in richer environments and, eventually, an emerging size is reached that maximizes host growth (see Supplementary Appendix C). As a consequence, for *w* values beyond a specific threshold, the host does not survive because growth (limited by its maximum value and nutrient availability) cannot overcome the increasing mortality due to dilution. Increasing crowding strength increases the emerging host size, which still ultimately saturates at the same value (see Supplementary Appendix B).

### Coevolution of Host and Virus

The coevolution of host and virus allows for wide regions of coexistence. Both plastic and non-plastic versions of the model predict similar qualitative behavior for nutrient and host density as a function of the dilution rate (Figure 2). As the dilution rate increases, both cases show an increasing nutrient availability and a slightly negative correlation with host availability. Viral density, however, shows a negative trend in the non-plastic case, whereas it remains approximately constant for the plastic model. From a quantitative point of view, host availability is larger for the plastic version of the model. For low to mid dilution rates, hosts in the plastic model show a much lower growth than the non-plastic case, which results in a lower viral burst size (Supplementary Figure 1). The consequent decrease in viral density decreases top-down pressure on the host, which ultimately reaches densities higher than those of the non-plastic model. Thus, viral density is lower for the plastic case for mid-to-low dilution rates, but vice versa for high dilution. For the latter regime, the non-plastic case shows higher nutrient densities, but for both cases the nutrient concentration saturates to *N_0* for high dilutions, which ultimately leads to similar growth rates and burst sizes but larger latent periods for the plastic case (Supplementary Figure 1). The emerging eclipse period is also longer for the latter case, whereas the maturation rate is smaller (Supplementary Figure 2).

**FIGURE 3:**
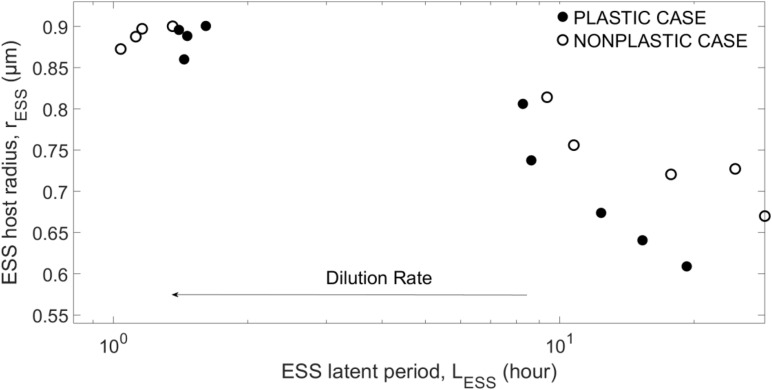
Evolutionary stable strategies (ESS) for the host and virus obtained for *N*_0_=10^−5^*moll*^−1^ and α=10*x*10^−9^*lcell*^−1^*d*^−1^, and different dilution rates.

As (Supplementary Figure 3) shows, the combinations (*L*_*ESS*_,*r*_*ESS*_) emerging from different replicates for a given dilution rate are clustered around a clear mean value, indicating that only one true combination of host size and latent period results from each environment. For a better visualization, we show in Figure 3 only the means for the evolving traits. The emergent host size and viral latent period show a negative correlation. For mid-to-low dilution rates, the same parametrization typically produces a smaller *r*_*ESS*_ for the plastic case than the non-plastic case, while *L*_*ESS*_ is smaller at lower dilution rates but becomes larger at higher dilution rates. At *w*=10*d*^−1^, *r*_*ESS*_ saturates to the host size that provides the highest growth for the fixed *N_0* (see Supplementary Appendix C), showing that the system is limited by external factors (i.e., nutrient input). Increasing N_0_ increases both *L*_*ESS*_ and *r*_*ESS*_, as well as the saturation threshold (see Supplementary Appendix C). Note that coexistence is not reached for *w* < 4*d*^−1^or *w* > 16*d*^−1^. In addition, the strength of the crowding effect tends to increase the emerging host size *r*_*ESS*_, but barely affects the emerging latent period *L*_*ESS*_ (see Supplementary Appendix B).

Host size plays two distinct roles in our model: it affects (i) host quality by influencing μ_*max*_ [Eq.(6)], and (ii) the number of infections by influencing the adsorption rate. In order to separate the influence of those effects on the emerging traits, we also check the emerging ESSs when the adsorption rate is fixed (i.e., *k*=3⋅10^−9^*lvirus*^−1^*d*^−1^). Our results do not change qualitatively when using a fixed adsorption rate (not shown).

### Comparison of Results for Coevolution Versus Single Evolution of Either Host or Virus

In order to understand the role of coevolution in the selection of the evolving traits (host size and viral latent period), we compare, respectively, the *r*_*ESS*_ and *L*_*ESS*_ emerging from coevolution with the emerging (i) *r*_*ESS*_ obtained when the host evolves alone and (ii) *L*_*ESS*_ obtained when the virus evolves with only one single host phenotype. For the latter, we use the expression $L_{E\,S\,S}=\frac{1}{w}+E\,\left(\mu \right)$, calculated analytically in Choua and Bonachela (2019).

#### Coevolution Versus Evolution of Host in the Absence of Virus

**FIGURE 4:**
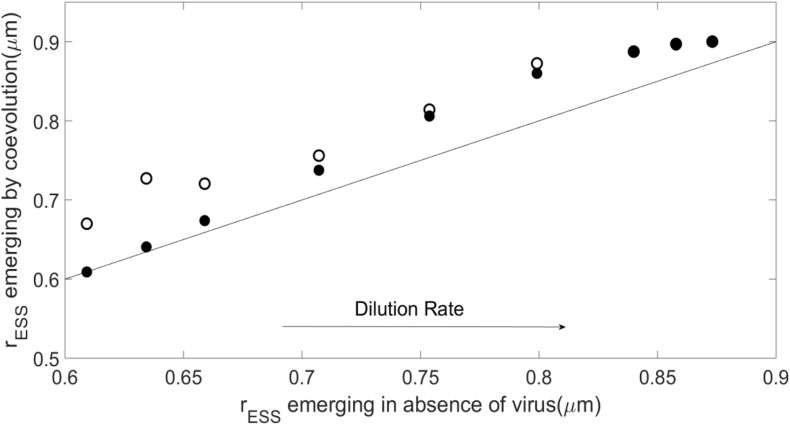
Evolutionary stable strategies (ESS) for the host obtained when it coevolves with the virus as compared with the host ESS in the absence of the virus for *N*_0_=10^−5^*moll*^−1^ and α=10^−8^*lcell*^−1^*d*^−1^, and different dilution rates. As in Figure 3, solid points represent the plastic case whereas empty points represent the non-plastic case, all above or on the 1:1 line.

Figure 4 shows that, as the dilution rates increases, the *r*_*ESS*_ resulting from the plastic case departs from the virus-free case, resulting in a larger host. The *r*_*ESS*_ in the non-plastic case shows larger host sizes than the plastic cases for all dilution rates, although both plastic and non-plastic cases converge to a similar value at high dilution rates. For low dilution rates, the emergent host size in the case of the plastic virus is similar to that without the virus. Supplementary Figure 4 shows that the resulting host growth rate follows a pattern similar to that described for host size. Host availability for the plastic case remains similar to the case without virus for most dilution rates, whereas the non-plastic virus keeps the host concentration lower than in the absence of the virus (Supplementary Figure 5).

#### Coevolution Versus Evolution of Virus

**FIGURE 5:**
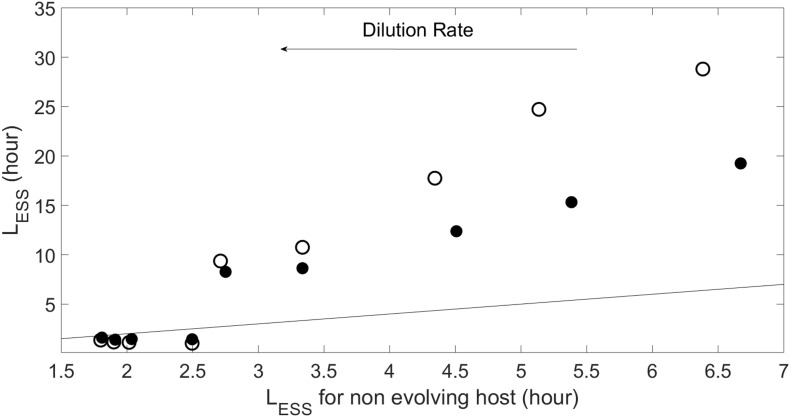
Evolutionary stable strategy (ESS) for the virus obtained when host evolves at *N*_0_=10^−5^*moll*^−1^ and α=10^−8^*lcell*^−1^*d*^−1^, and different dilution rates as compared to the viral ESS when the host does not evolve (i.e., *L* = 1/*w* + *E*). Symbols and line as in Figure 4.

As Figure 5 shows, coevolution leads to larger *L*_*ESS*_ than in the case of viral evolution with a fixed (i.e., non-evolving) host phenotype. This difference is more noticeable for the non-plastic case. Increasing the dilution rate (or strength of the crowding effect, see Supplementary Appendix B) reduces the difference between the with- and without-coevolution *L*_*ESS*_ for both plastic and non-plastic cases. In this regime the pattern actually inverts, the emergent latent period with coevolution becomes smaller than that with a non-evolving host, and the plastic case shows a *L*_*ESS*_ that is slightly larger than that of the non-plastic case.

## Discussion

Understanding the strategies that organisms use to survive under different ecological scenarios is key to understanding their role within their changeable ecosystem. Here, we studied the coevolution of a bacterial host and a phage. Viruses can impose a significant top-down pressure on their hosts, which may trigger different evolutionary responses in the latter. Our study can help understand which strategies are dominant for bacteria and viruses when coevolving under different scenarios, and the effect that viral plasticity (i.e., viral dependence on its host) has on these strategies. Typically, the virus drives the host to evolving defense mechanisms against phage infection, as repeatedly reported in the literature (Morgan and Koskella, 2011). In our case, the host evolves by changing its size, which has clear ecological implications: smaller hosts show a decreased rate of infection, but also a reduced maximal growth. The virus in turn can respond by adapting its infection time (i.e., latent period), which comes with the trade-off of either releasing fewer virions (shorter time) or losing opportunities of infection for its offspring (longer time).

In the absence of the virus, the emerging host strategy (*r*_*ESS*_) shows smaller sizes in environments with lower nutrient concentration, which result from lower dilution rates. This evolutionary strategy resonates with the fact that smaller microbes are better competitors when nutrient is scarce, like is the case for smaller phytoplankton (e.g., cyanobacteria) in oligotrophic waters (Mena et al., 2019).

For a same environment (i.e., for a fixed *w*), the phage pressure maintains the bacterial population to a lower level than in the absence of virus. Consequently, fewer bacteria compete for the nutrient and thus the host population grows faster, which ultimately results in larger emergent sizes. A logical expectation would be for the host to show, in the presence of the virus, a smaller size than in its absence, to avoid viral infection. Instead, the observed increase in host size indicates that the increase in metabolic rate associated to the larger size compensates for being a larger target for viral adsorption. This strategy also benefits the virus as it increases adsorption, and the improved host physiological state reduces latent period and increases burst size. Because a fixed adsorption rate did not alter our results, we conclude that the evolution of host size influences viral evolution mostly by altering host quality and, indirectly, host availability.

Increasing the dilution rate leads to larger host sizes (and therefore higher host quality) but lower host densities (i.e., lower host availability), which reveals a host quality-quantity trade-off. In nutrient-poor environments, the associated increased availability of the host with small size, which leads to small growth rates, i.e., low quality, tends to increase the latent period; this contrasts with past theory predicting that high host cell density favors phage with short latent period (Abedon, 1989). Thus, as we observe a decrease in the latent period even when the host availability decreases, we hypothesize that the lower host availability is compensated by the higher host quality, which appears to be the dominant factor in the selection of the latent period. This resonates with claims about the importance of host quality in determining the length of latent period when host density is high (Wang et al., 1996). Surprisingly, this quality-driven phenomenology is also shown by the non-plastic case. The reason is that, in spite of the viral traits being independent of the host growth rate in this case, evolutionary changes in host size determine viral trait values through their dependence on the maximum growth rate (e.g., Eq. 11), i.e., the non-plastic virus can produce more virions in a shorter time when host size increases. An important difference, thus, is that the latter occurs as a result of evolution, whereas viral plasticity is an ecological process.

Unexpectedly, the emerging latent period in the plastic case is shorter than in the non-plastic case for most dilution rates, and longer only for very high values of *w*. This result is in contrast with the definition of non-plastic virus we used here, as the latter shows the latent period expected at maximum growth rate (by definition the smaller possible value for *L*). Supplementary Figure 1 also shows that the growth rates reached by the host with the non-plastic virus are larger than those reached in the presence of the plastic virus, which should lead to a shorter latent period. The fact that the eclipse period is, as expected attending to host growth rate, longer in the plastic case and the maturation rate smaller (Supplementary Figure 2) means that the plastic virus spends less time to assembling virions, and does so at a slower rate. We hypothesize that, because here the non-plastic virus shows fewer hosts but of higher quality than the plastic virus, the lower *L* value for the latter might result from the effect of host availability on the emerging latent period, compensating the effect of the smaller host growth rate. With an abundant host, the strategy of the plastic virus is to lyse hosts earlier even if the resulting burst size is smaller. On the other hand, the lower amount of available hosts and the roughly one-order-of-magnitude difference in burst size requires from the non-plastic virus a longer latent period, to avoid host “overexploitation” (i.e., a Tragedy of the Commons scenario in which the virus kills the host and hence itself).

When the dilution rate is high, the burst size and host availability for both cases equalize, the only determining factor is the difference between host maximum growth rate (for the non-plastic virus traits) and realized growth rate (for the plastic virus traits) and, as a consequence, the non-plastic virus shows a shorter latent period than the plastic one. The differences in *L* and *B* between plastic and non-plastic virus are, however, small because the host growth rate for the former is close to its maximum. The crowding effect is an additional factor that regulates the bacterial population. The decrease in population resulting from crowding actually allows the host to reach the highest size that maximizes its growth rate for lower dilution rates, which can again be due to the lower competition for nutrient (see above). For sufficiently high α, the bacterial population is entirely regulated by the crowding effect, which explains the similar emergent size in the presence or absence of the virus.

Finally, the virus adapts to the evolutionary response of the host showing a *L*_*ESS*_ larger than that obtained when the host does not evolve, for both plastic and non-plastic viruses. Therefore, we conclude that coevolution slows down viral infection, the more so for a non-plastic virus. This delay and differences between plastic and non-plastic cases are amplified in nutrient-poor environments (i.e., for small dilution rates), where the host grows far from its maximal rate and the importance of plasticity is more observable. Without further empirical information, however, we cannot hypothesize the mechanisms underlying these strategies.

Our framework has a few important limitations. Although coevolutionary branching is a possibility that has been observed in laboratory and natural populations (Koskella and Brockhurst, 2014), we focused here on situations in which a single, well-defined ESS emerging for each simulated environment (evidenced by the clustered points obtained from the different replicates, i.e., each cluster can be represented by a single, well-defined mean value). Diversification can occur, for example, when a mutant virus adsorbs to only a limited subset of the total bacteria, i.e., it is a specialist (Weitz et al., 2005). In our case, however, viral phenotypes are all generalists [i.e., any viral mutant can infect any of the host phenotypes, shown to happen during coevolution (Hall et al., 2011)]. Although this “global” competition for hosts prevents a complete niche separation among viruses, we cannot exclude the possibility of evolutionary branching occurring in some of the discarded (surviving) replicates. Further, we discarded simulations in which either host or virus populations collapsed. The reason is that those simulations did not represent examples of “evolutionary suicide” but rather resulted from initial conditions for the traits that were outside the expected coexistence region of the parameter space [see (Choua et al., 2020)], and where evolution and/or plasticity did not reach the coexistence region before extinction. As a final remark, note that we have explored the role of one single environmental factor at a time: dilution rate, and nutrient input concentration (see Supplementary Appendix). Realistic conditions susceptible to be studied with our framework, from gut microbiome to phytoplankton populations, will necessitate of the inclusion of several abiotic (e.g., different nutrients) and biotic factors (e.g., grazers) varying simultaneously (Weitz et al., 2015; Pourtois et al., 2020).

## Conclusion

Although pathogens typically have detrimental effects on their hosts, in some cases the evolution of parasitic organisms may benefit the host by increasing host fitness (Morgan and Koskella, 2011). In our system, coevolution translates into the presence of virus fostering the emergence of larger host sizes that show larger growth potential, which is also beneficial for the virus as larger host growth rates improve also viral replication. We observe as a result an increased coexistence between host and phage. Our results, therefore, reveal an important aspect of the interaction between host and phage that should be considered when devising treatments to use phage against bacterial infections in either medical or environmental management contexts. This is especially relevant given the variability in host growth rate and sizes that is expected from bacterial infections, as well as the rapid evolutionary response that both bacteria and virus can show. Further, the predicted shift in host size due to the presence of the virus can have a cascading effect at larger scale in e.g., marine food webs. Cell size is a key trait for phytoplankton that influences the response of the lower trophic levels of the marine food web to climate change and carbon export to the deep ocean, among others (Finkel et al., 2009). Phytoplankton size, for example, influences mortality due to grazing, and therefore energy export to higher trophic levels. However, the dependence of phytoplankton maximum growth rate on cell size is non-monotonic, with smaller species following an increasing trend (qualitatively similar to that used here for *E. coli*) but larger species showing a negative correlation with cell size (Marañón et al., 2013). Our conclusions could therefore apply to species such as cyanobacteria and chlorophytes. Importantly, our theoretical framework is still applicable since the expectation is that similar dependence of viral performance on host physiology occur generically. Thus, our model could be easily modified to adapt it to other species and, ultimately, help to understand the processes involved in and determining the phytoplankton community structure, which in turn influences key biogeochemical cycles such as that of carbon, nitrogen, and phosphorus.

## Data Availability Statement

The code used to obtain the data supporting the conclusions of this article will be made available by the authors under request.

## Author Contributions

JAB and MH designed the research. MC conducted the research. MC and JAB wrote the manuscript with input from MH. All authors contributed to the article and approved the submitted version.

## Conflict of Interest

The authors declare that the research was conducted in the absence of any commercial or financial relationships that could be construed as a potential conflict of interest.

## References

[B1] Abedon S. T. (1989). Selection for bacteriophage latent period length by bacterial density: a theoretical examination. *Microb. Ecol.* 18 79–88. 10.1007/bf02030117 24196124

[B2] Abedon S. T. Herschler T. D. Stopar D. (2001). Bacteriophage latent-period evolution as a response to resource availability. *Appl. Environ. Microbiol.* 67 4233–4241. 10.1128/AEM.67.9.4233-4241.2001 11526028 PMC93152

[B3] Abedon S. T. Hyman P. Thomas C. (2003). Experimental examination of bacteriophage latent-period evolution as a response to bacterial availability. *Appl. Environ. Microbiol.* 69 7499–7506. 10.1128/aem.69.12.7499-7506.2003 14660403 PMC310036

[B4] Berg H. C. Purcell E. M. (1977). Physics of chemoreception. *Biophys. J.* 20 193–219. 10.1016/S0006-3495(77)85544-6 911982 PMC1473391

[B5] Birch E. W. Ruggero N. A. Covert M. W. (2012). Determining host metabolic limitations on viral replication via integrated modeling and experimental perturbation. *PLoS Comput. Biol.* 8:e1002746. 10.1371/journal.pcbi.1002746 23093930 PMC3475664

[B6] Bonachela J. A. Levin S. A. (2014). Evolutionary comparison between viral lysis rate and latent period. *J. Theor. Biol.* 345 32–42. 10.1016/j.jtbi.2013.12.006 24361326

[B7] Bremer H. Dennis P. P. (2008). Modulation of chemical composition and other parameters of the cell at different exponential growth rates. *EcoSal Plus* 2008:1128. 10.1128/Ecosal.5.2.3 26443740

[B8] Brown J. H. Gillooly J. F. Allen A. P. Savage V. M. West G. B. (2004). Toward a metabolic theory of ecology. *Ecology* 85 1771–1789. 10.1890/03-9000

[B9] Calendar R. L. Abedon S. T. (2005). *The Bacteriophages.* Oxford: Oxford University Press.

[B10] Chien A.-C. Hill N. S. Levin P. A. (2012). Cell size control in bacteria. *Curr. Biol.* 22 R340–R349. 10.1016/j.cub.2012.02.032 22575476 PMC3350639

[B11] Choua M. Bonachela J. A. (2019). Ecological and evolutionary consequences of viral plasticity. *Am. Natural.* 193 346–358. 10.1086/701668 30794445

[B12] Choua M. Heath M. R. Speirs D. C. Bonachela J. A. (2020). The effect of viral plasticity on the persistence of host-virus systems. *J. Theoret. Biol.* 498:110263. 10.1016/j.jtbi.2020.110263 32333976

[B13] De Paepe M. Taddei F. (2006). Viruses’ life history: towards a mechanistic basis of a trade-off between survival and reproduction among phages. *PLoS Biol.* 4:e193. 10.1371/journal.pbio.0040193 16756387 PMC1475768

[B14] Delbrück M. (1940). Adsoprtion of bacteriophage under various physiological conditions of the host. *J. Gen. Physiol.* 23 631–642. 10.1085/jgp.23.5.631 19873179 PMC2237952

[B15] Edwards K. F. Steward G. F. (2018). Host traits drive viral life histories across phytoplankton viruses. *Am. Natural.* 191 566–581. 10.1086/696849 29693441

[B16] Finkel Z. V. Beardall J. Flynn K. J. Antonietta Q. T. Rees A. V. Raven J. A. (2009). Phytoplankton in a changing world: cell size and elemental stoichiometry. *J. Plank. Res.* 32 119–137. 10.1093/plankt/fbp098 32665766

[B17] Füchslin H. P. Schneider C. Egli T. (2012). In glucose-limited continuous culture the minimum substrate concentration for growth, s(min), is crucial in the competition between the enterobacterium *Escherichia coli* and *Chelatobacter heintzii*, an environmentally abundant bacterium. *ISME J.* 6 777–789. 10.1038/ismej.2011.143 22030672 PMC3309354

[B18] Gallet R. Violle C. Fromin N. Jabbour-Zahab R. Enquist B. J. Lenormand T. (2017). The evolution of bacterial cell size: the internal diffusion-constraint hypothesis. *ISME J.* 11 1559–1568. 10.1038/ismej.2017.35 28375214 PMC5520153

[B19] Gnezda-Meijer K. Mahne I. Poljsak-Prijatelj M. Stopar D. (2006). Host physiological status determines phage-like particle distribution in the lysate. *FEMS Microb. Ecol.* 55 136–145. 10.1111/j.1574-6941.2005.00008.x 16420622

[B20] Golec P. Karczewska-Golec J. Łoś M. Wȩgrzyn G. (2014). Bacteriophage T4 can produce progeny virions in extremely slowly growing *Escherichia coli* host: comparison of a mathematical model with the experimental data. *FEMS Microbiol. Lett.* 351 156–161. 10.1111/1574-6968.12372 24386916

[B21] Hall A. R. Scanlan P. D. Buckling A. (2011). Bacteria-phage coevolution and the emergence of generalist pathogens. *Am. Natural.* 177 44–53. 10.1086/657441 21117957

[B22] Koskella B. Brockhurst M. A. (2014). Bacteria-phage coevolution as a driver of ecological and evolutionary processes in microbial communities. *FEMS Microbiol. Rev.* 38 916–931. 10.1111/1574-6976.12072 24617569 PMC4257071

[B23] Levin B. R. Stewart F. M. Chao L. (1977). Resource-limited growth, competition, and predation: a model and experimental studies with bacteria and bacteriophage. *Am. Natural.* 111 3–24. 10.1086/283134

[B24] Litchman E. Klausmeier C. A. Schofield O. M. Falkowski P. G. (2007). The role of functional traits and trade-offs in structuring phytoplankton communities: scaling from cellular to ecosystem level. *Ecol. Lett.* 10 1170–1181. 10.1111/j.1461-0248.2007.01117.x 17927770

[B25] Loferer-Krößbacher M. Klima J. Psenner R. (1998). Determination of bacterial cell dry mass by transmission electron microscopy and densitometric image analysis. *Appl. Environ. Microbiol.* 64 688–694. 10.1128/aem.64.2.688-694.1998 9464409 PMC106103

[B26] Marañón E. Cermeño P. López-Sandoval D. C. Rodríguez-Ramos T. Sobrino C. Huete-Ortega M. (2013). Unimodal size scaling of phytoplankton growth and the size dependence of nutrient uptake and use. *Ecol. Lett.* 16 371–379. 10.1111/ele.12052 23279624

[B27] Mena C. Reglero P. Hidalgo M. Sintes E. Santiago R. Martín M. (2019). Phytoplankton community structure is driven by stratification in the oligotrophic Mediterranean Sea. *Front. Microbiol.* 10:1698. 10.3389/fmicb.2019.01698 31396196 PMC6667633

[B28] Menge D. N. Weitz J. S. (2009). Dangerous nutrients: evolution of phytoplankton resource uptake subject to virus attack. *J. Theor. Biol.* 257 104–115. 10.1016/j.jtbi.2008.10.032 19068219

[B29] Monod J. (1949). The growth of bacterial cultures. *Annu. Rev. Microbiol.* 3 371–394. 10.1146/annurev.mi.03.100149.002103

[B30] Morgan A. D. Koskella B. (2011). “6 – Coevolution of host and pathogen,” in *Genetics and Evolution of Infectious Disease*, ed. Tibayrenc M. (London: Elsevier), 147–171.

[B31] Nikaido H. Vaara M. (1985). Molecular basis of bacterial outer membrane permeability. *Microbiol. Rev.* 49 1–32. 10.1128/mmbr.49.1.1-32.1985 2580220 PMC373015

[B32] Pourtois J. Tarnita C. Bonachela J. A. (2020). Impact of lytic phages on phosphorus- versus nitrogen-limited marine microbes. *Front. Microbiol.* 11:221. 10.3389/fmicb.2020.00221 PMC7047511 32153528

[B33] Rabinovitch A. Fishov I. Hadas H. Einav M. Zaritsky A. (2002). Bacteriophage T4 development in *Escherichia coli* is growth rate dependent. *J. Theor. Biol.* 216 1–4. 10.1006/jtbi.2002.2543 12076123

[B34] Rabinovitch A. Hadas H. Einav M. Melamed Z. Zaritsky A. (1999). Model for bacteriophage T4 development in *Escherichia coli*. *J. Bacteriol.* 181 1677–1683. 10049403 10.1128/jb.181.5.1677-1683.1999 PMC93561

[B35] Ramanculov E. Young R. (2001). Genetic analysis of the T4 holin: timing and topology. *Gene* 265 25–36. 10.1016/s0378-1119(01)00365-1 11255004

[B36] Schulze K. L. Lipe R. S. (1964). Relationship between substrate concentration, growth rate, and respiration rate of *Escherichia coli* in continuous culture. *Archiv. Mikrobiol.* 48 1–20. 10.1007/bf00406595 14196722

[B37] Schwartz M. (1976). The adsorption of coliphage lambda to its host: effect of variations in the surface density of receptor and in phage-receptor affinity. *J. Mol. Biol.* 103 521–536. 10.1016/0022-2836(76)90215-1 181582

[B38] Shestopaloff Y. K. (2016). Interspecific allometric scaling of unicellular organisms as an evolutionary process of food chain creation. *arXiv* [Preprint]. arXiv:1611.09824.

[B39] Thyrhaug R. Larsen A. Thingstad T. F. Bratbak G. (2003). Stable coexistence in marine algal host-virus systems. *Mar. Ecol. Progr. Ser.* 254:25451.

[B40] Tilman D. (1982). Resource competition and community structure. *Monogr. Populat. Biol.* 17 1–296. 7162524

[B41] Wang I.-N. (2006). Lysis timing and bacteriophage fitness. *Genetics* 172 17–26. 10.1534/genetics.105.045922 16219778 PMC1456144

[B42] Wang I.-N. Dykhuizen D. Slobodkin L. (1996). The evolution of phage lysis timing. *Evolut. Ecol.* 10 545–558. 10.1007/BF01237884

[B43] Wang I.-N. Smith D. L. Young R. (2000). Holins: the protein clocks of bacteriophage infections. *Annu. Rev. Microbiol.* 54 799–825. 10.1146/annurev.micro.54.1.799 11018145

[B44] Wang Z. Goldenfeld N. (2010). Fixed points and limit cycles in the population dynamics of lysogenic viruses and their hosts. *Phys. Rev. E Statist. Nonlin. Soft Matter Phys.* 82(1 Pt 1):011918. 10.1103/physreve.82.011918 20866659

[B45] Ward B. A. Marañón E. Sauterey B. Rault J. Claessen D. (2017). The size dependence of phytoplankton growth rates: a trade-off between nutrient uptake and metabolism. *Am. Natural.* 189 170–177. 10.1086/689992 28107051

[B46] Weitz J. S. Dushoff J. (2008). Alternative stable states in host–phage dynamics. *Theoret. Ecol.* 1 13–19. 10.1007/s12080-007-0001-1

[B47] Weitz J. S. Hartman H. Levin S. A. (2005). Coevolutionary arms races between bacteria and bacteriophage. *Proc. Natl. Acad. Sci. U.S.A.* 102 9535–9540. 10.1073/pnas.0504062102 15976021 PMC1172273

[B48] Weitz J. S. Stock C. A. Wilhelm S. W. Bourouiba L. Coleman M. L. Buchan A. (2015). A multitrophic model to quantify the effects of marine viruses on microbial food webs and ecosystem processes. *ISME J.* 9 1352–1364. 10.1038/ismej.2014.220 25635642 PMC4438322

[B49] White R. Chiba S. Pang T. Dewey J. S. Savva C. G. Holzenburg A. (2011). Holin triggering in real time. *Proc. Natl. Acad. Sci. U.S.A.* 108:798. 10.1073/pnas.1011921108 21187415 PMC3021014

[B50] Wirtz K. W. (2002). A generic model for changes in microbial kinetic coefficients. *J. Biotechnol.* 97 147–162. 10.1016/s0168-1656(02)00064-0 12067521

[B51] You L. Suthers P. Yin J. (2002). Effects of *Escherichia coli* physiology on growth of phage T7 in vivo and in silico. *J. Bacteriol.* 184 1888–1894. 10.1128/jb.184.7.1888-1894.2002 11889095 PMC134924

[B52] Young R. Bläsi U. (1995). Holins: form and function in bacteriophage lysis. *FEMS Microbiol. Rev.* 17 191–205. 10.1016/0168-6445(94)00079-4 7669346

