# Supplementary material for: Evolutionarily Stable Coevolution Between a Plastic Lytic Virus and Its Microbial Host
Source: Front Microbiol. 2021 May 20;12:637490. doi: 10.3389/fmicb.2021.637490 (PMC8172972; doi:10.3389/fmicb.2021.637490)
Supplement: Supplementary file 1 [file Data_Sheet_1.pdf]

Evolutionarily stable coevolution between a plastic lytic virus and its microbial host

Appendix

Melinda CHOUA[1], Michael R. HEATH[1] and Juan A. BONACHELA[2]*

[1] *Marine Population Modelling Group, Department of Mathematics and Statistics, University of Strathclyde, Glasgow, Scotland, United Kingdom.*

[2] *Department of Ecology, Evolution and Natural Resources, Rutgers University, New Brunswick, New Jersey, United States.*

A. Emergent host size in the absence of virus and crowding effect

In a simple system where the virus and crowding effects are neglected (i.e. $V = \alpha = 0$), the bacterial dynamics are described by :

$$\frac{dC_i(t)}{dt} = (\mu_i - w)C_i \qquad \text{(A.1)}$$

$$\frac{dN}{dt} = w(N_0 - N) - \frac{1}{Y}\sum_i \mu_i(N)\, C_i \qquad \text{(A.2)}$$

Such simplified system enables reaching analytical stationary states for both the ecological and evolutionary dynamics. For the ecological stationary state, we first focus on one single phenotype and calculate the solution to the equations $dN/dt = dC/dt = 0$ given by:

$$C_{st} = Y(N_0 - N_{st}) \qquad \text{(A.3)}$$

$$N_{st} = \frac{w\, K_n}{\mu_{max} - w} \qquad \text{(A.4)}$$

For the evolutionary stationary state, we conduct the invasion analyses by studying the stability of this resident bacteria-nutrient system that, after reaching equilibrium, is perturbed by an invading bacterial phenotype (or, equivalently, i.e. bacterial strain with a different size and associated metabolic rates) that challenges the resident. Because the

mutant is initially rare, both N and C_R stay at their stationary values (i.e. $C = C_{R_{st}} = C_{st}$ and $N = N_{R_{st}} = N_{st}$) and the system dynamics is reduced to the following equation:

$$\frac{dC_M(t)}{dt} = (\mu_M - w)C_M \qquad \text{(A.1)}$$

The stability of the system is thus given by the sign of the eigenvalues λ obtained by solving the characteristic equation given by $\mu_M - w - \lambda = 0$, where λ is the associated eigenvalue. The system is stable (i.e. the perturbation fades away) if $\lambda < 0$, i.e. if $\mu_M < w$, which leads to:

$$\mu_M = \mu_{max_M} \frac{N_{st}}{K_{n_M} + N_{st}} < w \iff \mu_{max_M} N_{st} < K_{n_M} w + N_{st} w$$

$$N_{st}\left(\mu_{max_M} - w\right) < K_{n_M} w \iff N_{st} < \frac{K_{n_M} w}{\left(\mu_{max_M} - w\right)}$$

$$\underset{using\ Eq\ (A.4)}{\Longleftrightarrow} N_{st_R} < N_{st_M}$$

Therefore, the bacterial resident that resists any invasion shows a size such that minimizes nutrient requirement, in agreement with classic competition theory (Tilman 1982). Thus, we look for the value of r such that:

$$\frac{dN_{st}}{dr} = \frac{dN_{st}}{d\mu_{max}} \cdot \frac{d\mu_{max}}{dr} = 0 \qquad \text{(A.6)}$$

Because $\frac{d\mu_{max}}{dr}$ equals zero only when $r = 0$, we thus focus on $\frac{dN_{st}}{d\mu_{max}} = 0$. Using Eq. (A.4), we obtain 2 extrema, and the minimum ($d^2N_{st}/dr^2 > 0$) is given by:

$$\mu_{max} = \frac{3\ \mu_{ref} - \sqrt{5\ \mu_{ref}^{\ 2} - 4\ \mu_{ref}\ w}}{2} \qquad \text{(A.7)}$$

Using the equation that links μ_{max} and r (i.e. Eq. (6)), we extract r_{ESS}:

$$r_{ESS_{V=0,\alpha=0}} = \sqrt[3]{\frac{3}{4\pi\ Conv_1} . 10^{\frac{log_{10(\mu max_{ESS}/Conv2)-p}}{f}}} \quad (A.8)$$

Consequently, r_{ESS} depends only on w in the absence of the virus and the crowding effect. Note that Eq. (A.7) is feasible only for $w < \frac{5}{4}\mu_{ref} = 40.5\ d^{-1}$, which is always the case here. Indeed, for dilution rate above values equal to $\mu(\tilde{r}\ )$ appearing in Table C.1, any bacterial phenotype goes to extinction (i.e. Eq.(A.1) negative due to $\mu < w$).

B. Effect of crowding on the emerging host size

In cases where the crowding effect is considered (i.e. $\alpha \neq 0$), the evolutionary stationary state of the host in the absence of the virus is calculated numerically using the genetic algorithm described in the main text. The resulting $r_{ESS_{V=0,\alpha\neq0}}$ is then collected for different w, and compared to (i) $r_{ESS_{V=0,\alpha=0}}$ in order to see the effect of crowding effect on the host size evolution, and (ii) the r_{ESS} obtained during coevolution with the plastic and nonplastic virus (see Fig. B.1).

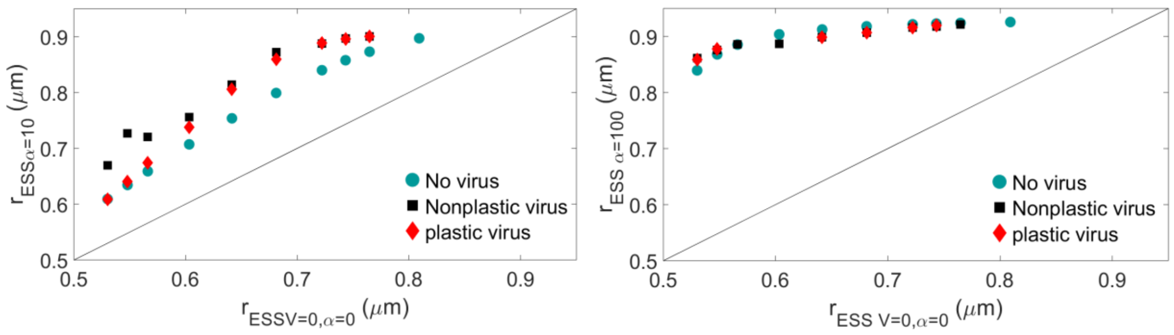

Figure B.1: Evolutionary stable strategy (ESS) for the host obtained when it coevolves with the virus at $N_0 = 10^{-5}\ mol\ l^{-1}$ for different dilution rates compared with the ESS of the host in absence of virus and $\alpha = 0$. The black line represents the line in which the y axis equals the x axis. Left: $\alpha = 10^{-8}\ l\ cell^{-1}d^{-1}$. Right: $\alpha = 10^{-7}\ l\ cell^{-1}d^{-1}$

The emerging r_{ESS} increases with the dilution rate and crowding strength, and eventually converges to $\tilde{r}$ (see Appendix C and Table C.1). Convergence to this value is faster in the presence of viruses, and as the crowding effect increases. At high α values, the presence of the virus does not make a significant difference for the evolutionary strategy of the host, and the effect of plasticity is not easily discernible with this parametrization.

For all crowding strengths α, there exists a negative correlation between the emergent host size and viral latent period. The difference between the plastic and nonplastic cases is noticeable at low crowding strength but decreases as α increases; for high-enough α, both cases produce overlapping r_{ESS} vs L_{ESS} combinations (see appendix B). Although the crowding effect affects the emerging host size, it does not influence the emerging latent period of the virus.

$w\ (d^{-1})$	4	5	6	8	10	11	14	15	16
PLASTIC	●	★	■	♦	▼	▲	◀	▶	✸
NONPLASTIC	○	☆	□	◇	▽	△	◁	▷	✡

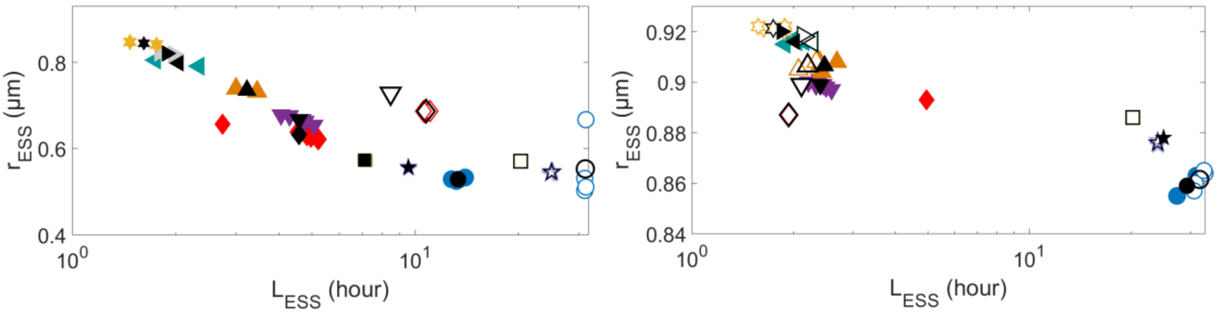

Figure B.2: Evolutionary stable strategy (ESS) for the host and virus obtained at $N_0 = 10^{-5}\ mol\ l^{-1}$ *for different dilution rates and crowding effect. Left:* $\alpha = 0$. *Right:* $\alpha = 10^{-7}\ l\ cell^{-1} d^{-1}$. *Symbols as in Supplementary Figure 3 (see section D).*

C. *Effect of nutrient input on the emerging traits*

Both L_{ESS} and r_{ESS} increase as the input nutrient concentration increases, r_{ESS} saturates as the dilution rates increases.

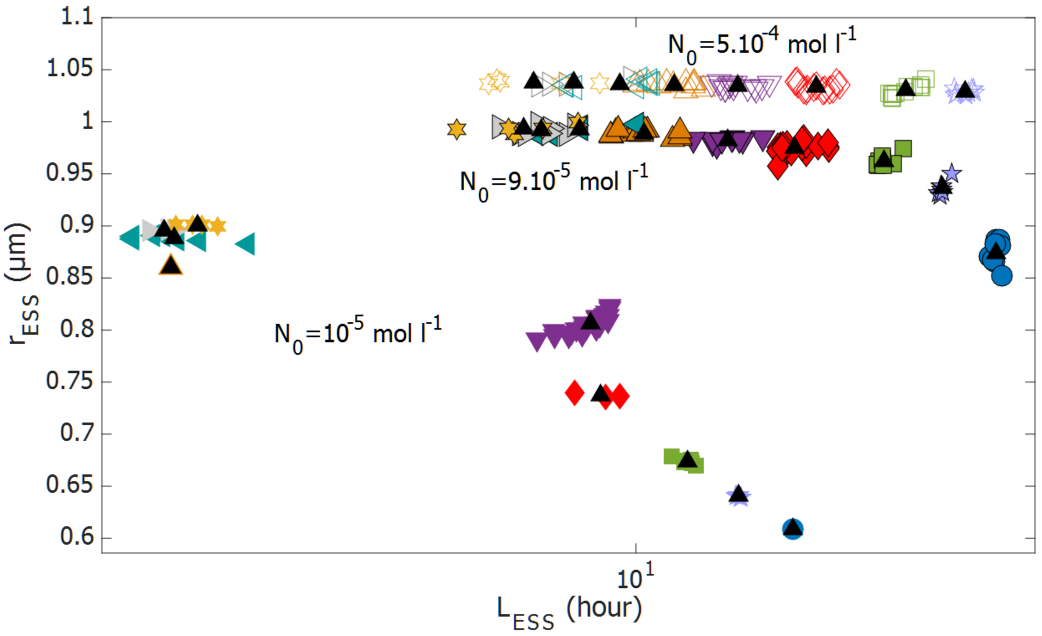

Figure C.1: Evolutionary stable strategy (ESS) for the host and virus obtained for different dilution rates and different input nutrient concentrations for the plastic case at $\alpha = 10^{-8}\, l\, cell^{-1} d^{-1}$. *The symbols with no contour represent the* $N_0 = 10^{-5}\, mol\, l^{-1}$ *case; symbols with black contour, the* $N_0 = 9 \cdot 10^{-5}\, mol\, l^{-1}$*case; and empty symbols, the* $N_0 = 5 \cdot 10^{-4}\, mol\, l^{-1}$ *case. Symbols as in Supplementary Figure 3 (see section D).*

Each N_0 case saturates close to the host size that provides the highest growth for that given N_0 (i.e. the value for r that provides the highest growth rate if $N \to N_0$, see Table C.1 for values). Indeed, sizes higher than those in Table C.1 shows a smaller growth rate at $N = N_0$ due to the relationship in Eq. (7).

Table C.1: Values of the host radius that shows the highest growth rate when the nutrient concentration in the chemostat is at its highest possible concentration (i.e. $N = N_0$*). Values for r represent averages over replicates, rounded to the second decimal figure.*

$N_0\ [mol\ l^{-1}]$	10^{-5}	$9 \cdot 10^{-5}$	$5 \cdot 10^{-4}$
$\mu(\tilde{r})\ [d^{-1}]$	14.8	15.9	16.6
$\tilde{r}\ [\mu m]$	0.93	1.00	1.04

D. Supplementary figures

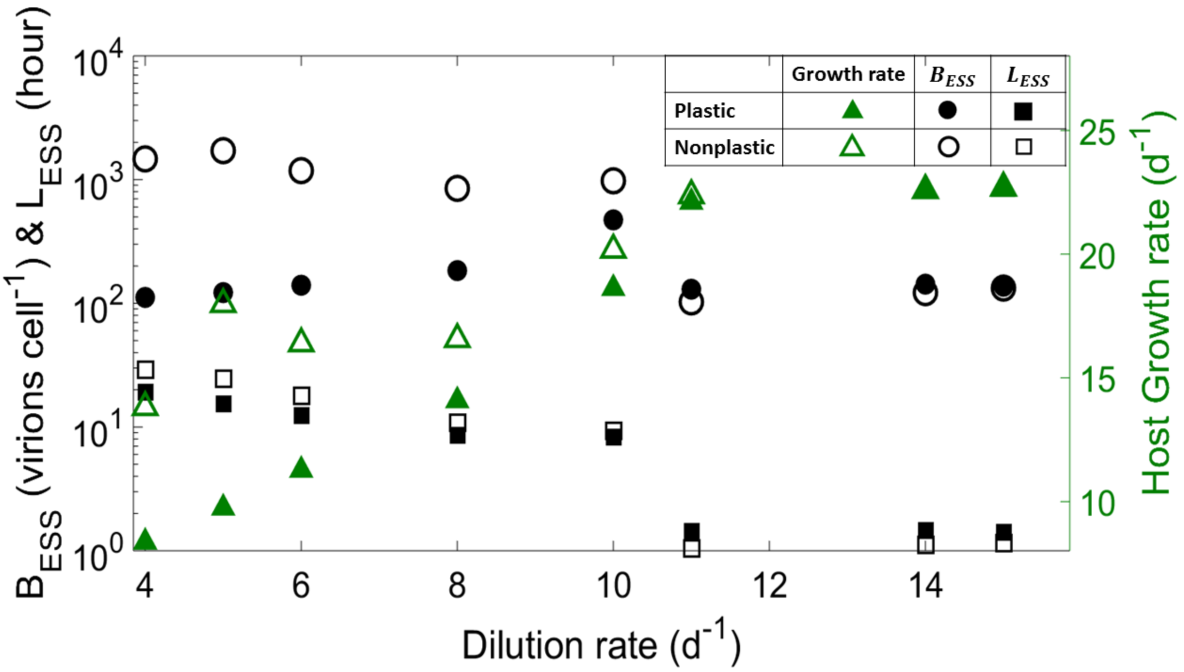

Supplementary Figure 1: Evolutionary stable strategy (ESS) for the virus and its associated emerging burst size (left axis), and the host growth rate (right axis) as a function of the dilution rate.

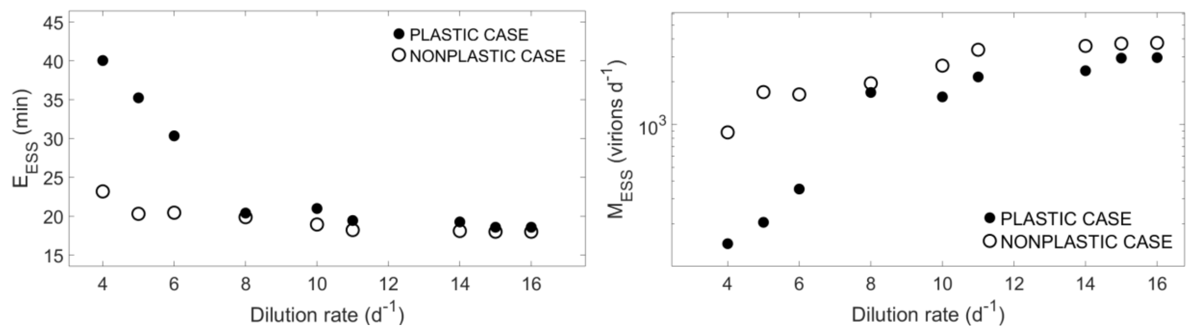

Supplementary Figure 2: Evolutionary stable strategy (ESS) for the eclipse period (left) and maturation rate (right) as a function of the dilution rate.

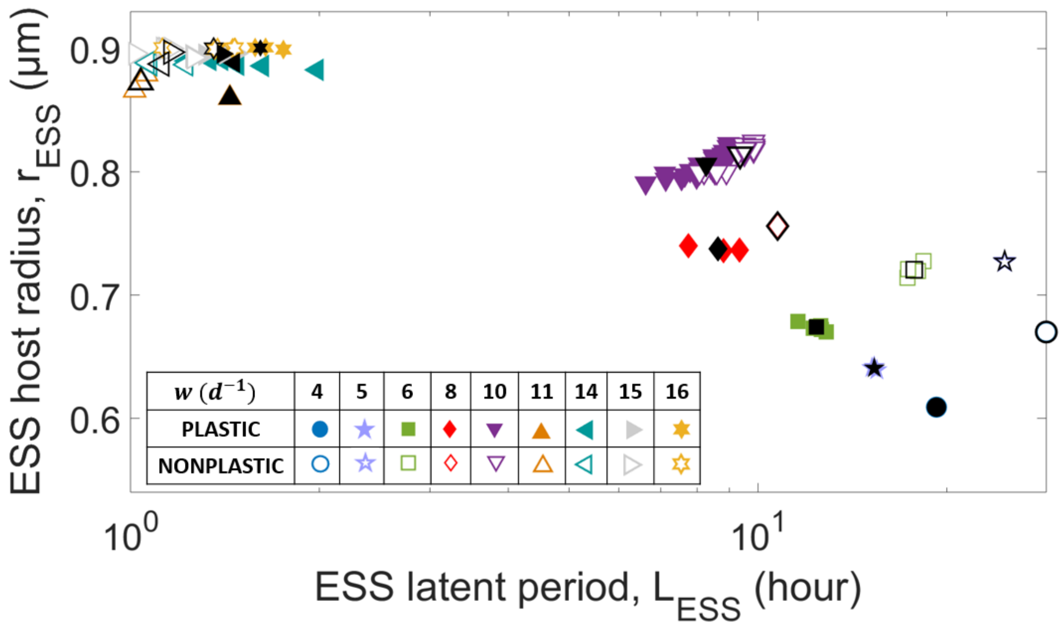

Supplementary Figure 3: Cluster of evolutionary stable strategy (ESS) for the host and virus at $N_0 = 10^{-5}\ mol\ l^{-1}$ *and* $\alpha = 10^{-8}\ l\ cell^{-1} d^{-1}$*. Means for each dilution rate are shown in black.*

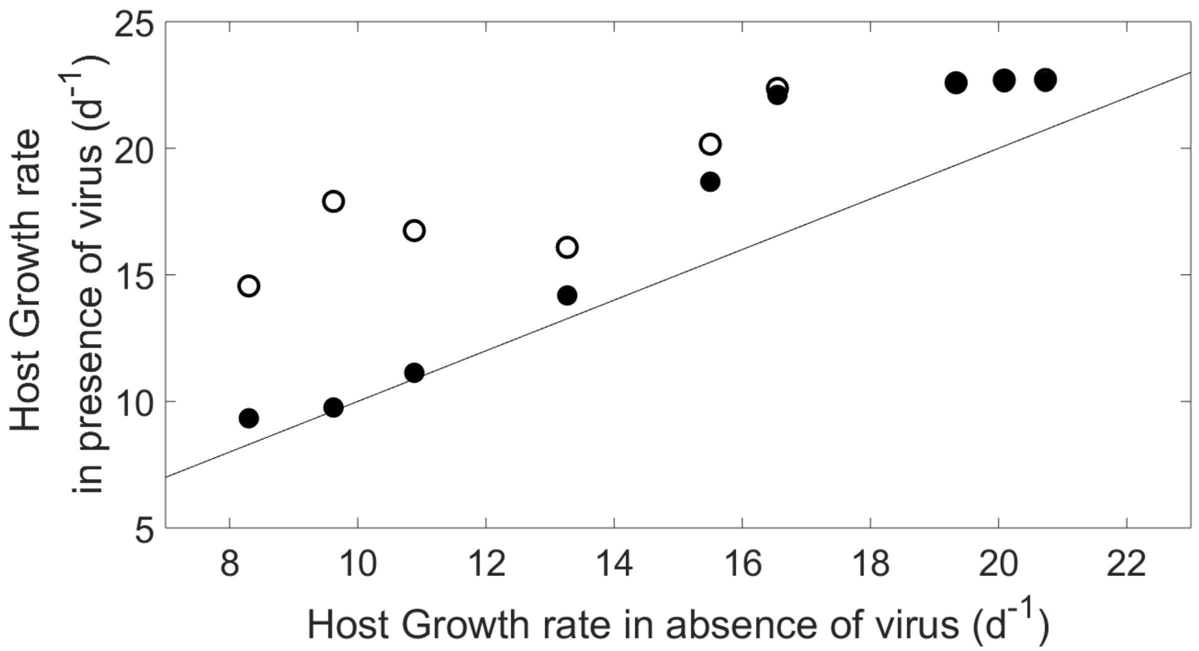

Supplementary Figure 4: Comparison between the host growth rate in the absence and in the presence of the virus, the latter for both plastic (solid dots) and nonplastic version of the model (empty dots). The presence of the virus leads to higher growth rates, with the plastic version showing lower rates that are comparable to those without the virus for mid-to-low dilution rates.

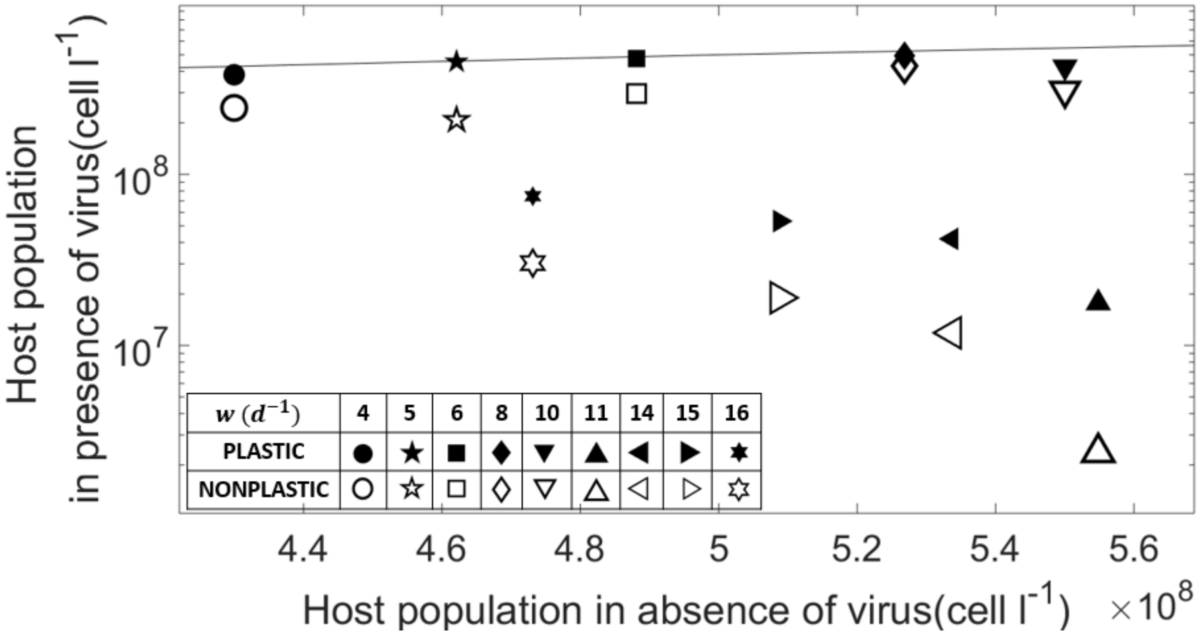

Supplementary Figure 5: Host availability in the presence of viruses as a function of the host availability without viruses. As indicated by Fig. 2, the host population co-evolving with a plastic virus shows larger concentrations than in the presence of a nonplastic virus. The host population shows a larger concentration when evolving without the virus, and a much narrower range of variation for the dilution rates sampled here.

References

Tilman, D. 1982. "Resource competition and community structure." *Monographs in population biology* 17:1-296.
